# Structural Health Monitoring of Fatigue Cracks for Steel Bridges with Wireless Large-Area Strain Sensors

**DOI:** 10.3390/s22145076

**Published:** 2022-07-06

**Authors:** Sdiq Anwar Taher, Jian Li, Jong-Hyun Jeong, Simon Laflamme, Hongki Jo, Caroline Bennett, William N. Collins, Austin R. J. Downey

**Affiliations:** 1 Department of Civil, Environmental and Architectural Engineering, The University of Kansas, Lawrence, KS 66045, USA; sdiq.anwar@ku.edu (S.A.T.); crb@ku.edu (C.B.); william.collins@ku.edu (W.N.C.); 2 Department of Civil, Architectural Engineering and Mechanics, The University of Arizona, Tucson, AZ 85721, USA; jhjeong@email.arizona.edu (J.-H.J.); hjo@email.arizona.edu (H.J.); 3 Department of Civil, Construction and Environmental Engineering, Iowa State University, Ames, IA 50011, USA; laflamme@iastate.edu; 4 Department of Electrical and Computer Engineering, Iowa State University, Ames, IA 50011, USA; 5 Department of Mechanical Engineering, University of South Carolina, Columbia, SC 29208, USA; austindowney@sc.edu; 6 Department of Civil and Environmental Engineering, University of South Carolina, Columbia, SC 29208, USA

**Keywords:** structural health monitoring, fatigue crack, soft elastomeric capacitor, wireless sensors, large-area strain sensor, civil infrastructure, steel bridges, generalized Morse wavelet, peak detection, traffic loads

## Abstract

This paper presents a field implementation of the structural health monitoring (SHM) of fatigue cracks for steel bridge structures. Steel bridges experience fatigue cracks under repetitive traffic loading, which pose great threats to their structural integrity and can lead to catastrophic failures. Currently, accurate and reliable fatigue crack monitoring for the safety assessment of bridges is still a difficult task. On the other hand, wireless smart sensors have achieved great success in global SHM by enabling long-term modal identifications of civil structures. However, long-term field monitoring of localized damage such as fatigue cracks has been limited due to the lack of effective sensors and the associated algorithms specifically designed for fatigue crack monitoring. To fill this gap, this paper proposes a wireless large-area strain sensor (WLASS) to measure large-area strain fatigue cracks and develops an effective algorithm to process the measured large-area strain data into actionable information. The proposed WLASS consists of a soft elastomeric capacitor (SEC) used to measure large-area structural surface strain, a capacitive sensor board to convert the signal from SEC to a measurable change in voltage, and a commercial wireless smart sensor platform for triggered-based wireless data acquisition, remote data retrieval, and cloud storage. Meanwhile, the developed algorithm for fatigue crack monitoring processes the data obtained from the WLASS under traffic loading through three automated steps, including (1) traffic event detection, (2) time-frequency analysis using a generalized Morse wavelet (GM-CWT) and peak identification, and (3) a modified crack growth index (CGI) that tracks potential fatigue crack growth. The developed WLASS and the algorithm present a complete system for long-term fatigue crack monitoring in the field. The effectiveness of the proposed time-frequency analysis algorithm based on GM-CWT to reliably extract the impulsive traffic events is validated using a numerical investigation. Subsequently, the developed WLASS and algorithm are validated through a field deployment on a steel highway bridge in Kansas City, KS, USA.

## 1. Introduction

Repetitive service loads produce fatigue cracks in metallic civil infrastructures, e.g., steel bridges, which pose great threats to their structural integrity and may lead to catastrophic failures. The current de facto method for detecting and monitoring fatigue cracks is visual inspection carried out by trained inspectors, which may be inaccurate and susceptible to errors due to the small size and randomness of fatigue cracks [1]. To improve accuracy and prevent catastrophic failures, a more effective and efficient technique for monitoring those fatigue cracks is critical to ensure timely actions. To this end, structure health monitoring (SHM) has attracted significant attention since it can provide continuous, reliable, and accurate monitoring at a lower cost. In particular, by integrating sensors for measuring structural responses, data processing and modeling algorithms for diagnosing structural conditions, and presenting a prognosis of future status, SHM has shown great success in global structural assessment through system/modal identification [2], model updating [3], and input and state estimation [4] using acceleration and strain measurements. However, SHM in field applications, particularly for local damage such as fatigue crack monitoring, is still lacking due to the lack of appropriate sensors, their integration with energy-efficient wireless sensing platforms for long-term autonomous monitoring, and effective algorithms for the prognosis and diagnosis of local fatigue damage. This paper focuses on this issue by proposing an integrated wireless large-area strain sensor network with tailored signal processing algorithms for the long-term fatigue crack monitoring of steel bridges.

SHM based on traditional sensing technologies, e.g., acceleration and strain, has been extensively studied, playing important roles in system and modal identification, as well as structural and safety evaluations for civil structures. One early example based on wired sensors is the health monitoring of the Hakucho Suspension Bridge in Japan, for which modal properties of the bridge were identified and studied based on ambient vibration measurements [5]. Other examples include the monitoring of several long-span cable-supported bridges in Hong Kong, China [6], and the cable-stayed Bill Emerson Memorial Bridge in Cape Girardeau, Missouri, USA [7]. These studies were based on wired sensors, which have limitations including difficulties associated with installation, high cost, and data inundation. In recent years, wireless smart sensors for SHM have received considerable attention to overcome those limitations. A pioneer in this area is a wireless sensor network designed based on MicaZ mote measuring four channels of accelerations in two directions [2]. As a proof of concept, the sensor network was installed on the Golden Gate Bridge in the USA. Although successful modal identification was obtained based on the measured data, the wireless sensor network was only designed to measure accelerations and had network scalability issues due to data transmission efficiency [8]. Later, a flexible wireless smart sensor framework based on the iMote2 was proposed [9], and a full-scale sensor network was deployed on a cable-stayed bridge, the 2nd Jindo Bridge, in southern South Korea for long-term monitoring [10]. Recently, this framework was extended to a new generation sensing platform, termed the Xnode, which is deployed with the FreeRTOS real-time operating system (RTOS). Xnode is capable of event-triggered sensing to only capture significant events while saving power. It also offers a flexible interface to external sensors, long-range communication for wireless data retrieval, and cloud storage [8,11,12]. The above advancements in wireless sensor networks and signal processing algorithms have enabled robust, efficient, and effective monitoring of global modal properties for civil infrastructure. However, long-term monitoring of local structural damage such as fatigue cracks, which require structural responses measured at the local scale, has received less attention.

In SHM, structural surface strain changes can be captured using strain sensors, such as metal foil strain gauges and fiber optic sensors, which are considered as point and distributed one-dimensional sensors, respectively. Applications include crack detection for aircraft structures using strain gauges [13] and monitoring strain and temperature using fiber optic sensors in bridges [14,15,16,17]. When a fatigue crack occurs, strain measurements can be effective for crack detection and monitoring because of the sensitivity of strain change to the opening and closing of the crack, especially when the strain sensors are installed close to the crack [18]. However, the point and distributed one-dimensional sensors may not provide adequate information for fatigue crack monitoring since their small sizes hinder their ability to cover an adequate surface of fatigue crack prone areas [18,19,20,21]. Moreover, the limited ductility of those sensors causes them to fail under the extreme strain demand due to crack formation, and using a dense array of them to cover large areas to provide adequate surface coverage may be impractical and expensive [18,19,20,21].

To address the above issues, various large-area sensors were proposed to measure strain over a large area. For instance, strain sensing sheets which can cover large areas were proposed for damage and crack detections [19,22,23], in which laboratory experiments and a pedestrian bridge were used to validate the proposed sensors for damage and crack detections. An antenna sensor was developed for monitoring fatigue crack growth and opening in a compact tension (C(T)) specimen under fatigue loading [24]. Further development of the antenna sensor led to a wireless patch antenna sensor [25] for strain and crack sensing, which was validated through laboratory experiments. A carbon nanotube (CNT) sensor was studied for monitoring and detecting fatigue cracks in metal structures utilizing laboratory tests [26], and a wireless stretchable sensor network [27], including piezoelectric, strain gauge, and resistive temperature sensors, was integrated to obtain measurements over an aerospace composite wing. Of interest to this research, a soft elastomeric capacitive (SEC) sensor was proposed to monitor strain over a large area [20]. The SEC was investigated for the identification of modal parameters, natural frequency, and mode shape for both steel and concrete specimens in laboratory settings [28]. Overall, large-area strain sensing provided promising results in laboratory environments, but validations for the wireless long-term monitoring of fatigue crack under realistic traffic loadings in the field have been limited.

Due to its high stretchability, flexibility, and ability to measure large-area strain, the SEC is especially suited for fatigue crack monitoring. The performance of SEC was first investigated in [29] for the detection and localization of low-cycle fatigue cracks using laboratory C(T) specimens. A robust data processing approach for harmonic loading, a crack growth index (CGI), was later proposed in [18] for monitoring high-cycle fatigue cracks, generated using a loading protocol that maintains a constant range of stress intensity to restrict plastic deformation at the crack tip. The above studies proved the SEC’s capability in monitoring fatigue cracks under idealized harmonic fatigue load cycles and a constant crack propagation rate. Under more realistic cases, further investigation was performed [30] for figure crack monitoring using the proposed CGI and a large-scale laboratory bridge girder, demonstrating the potential of the SEC in monitoring the fatigue crack.

The above studies demonstrated the effectiveness of the large-area strain sensor, SEC, for fatigue crack monitoring in the laboratory environment. However, the laboratory studies utilized a wired data acquisition system and were based on simplifying the traffic loading as a harmonic function, whereas real traffic-induced bridge responses consist of impulsive signals. To fill these gaps in the knowledge and transfer this technology from the laboratory to the field for long-term bridge fatigue crack monitoring, this paper presents two critical novelties: (1) by integrating the SEC with the Xnode sensing platform, a wireless large-area strain sensor (WLASS) is created to wirelessly collect the large-area strain data to support fatigue crack monitoring; (2) an effective automated algorithm is developed based on wavelet transform to process the traffic-induced bridge response data consisting of numerous impulsive peak events for monitoring fatigue crack growth. The remainder of this paper is organized as follows. Section 2 presents the problem statement and the key mechanism for sensing fatigue crack growth. Section 3 describes the main proposed methodology including both the hardware and algorithm aspects of the WLASS. Section 4 validates the proposed algorithm through a numerical example. Section 5 describes a field deployment of the WLASS and presents the monitoring data to validate the proposed methodology. Section 6 summarizes the main conclusions of this study.

## 2. Problem Statement

**Figure 1:**
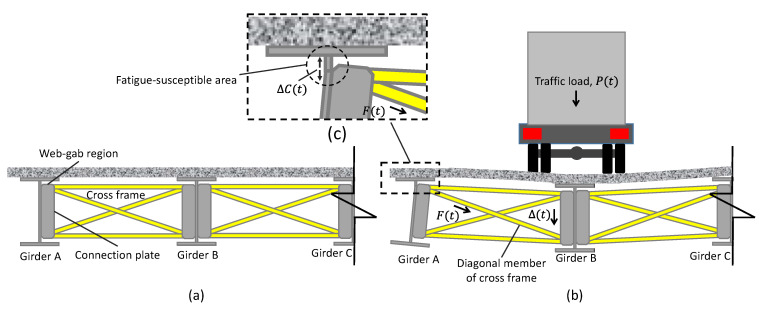
Distortion-induced fatigue of steel bridges: (**a**) girders with no traffic loading, and (**b**) girders with traffic loading, and (**c**) fatigue crack developed at the web-gab region.

This paper is motivated by the pressing need for reliable SHM of fatigue cracks for civil infrastructures. In particular, distortion-induced fatigue cracks in steel bridges are the focus of this study. Distortion-induced fatigues typically happen at web-gap regions of steel girder bridges where the girder web, flange, and connection plates meet. Figure 1a,b illustrate the mechanism of the distortion-induced fatigue in the girder web of a steel bridge. The steel bridge consists of cross-frames connected with girders by transverse connection plates. For most steel girder bridges built before the mid-1980s in the United States, the connection plates are not welded to the flanges, creating web-gap regions located between the connection plate and the flanges. As shown in Figure 1b, under traffic loading, $P\left(t\right)$, adjacent girders face differential displacement, $\Delta \left(t\right)$, leading to out-of-plane force , F(t), being exerted on the girder web by the cross-frames through the transverse connection plates. Since the web-gab is flexible, the out-of-plane force, $F\left(t\right)$, of the cross-frame causes distortion-induced fatigue and further leads to fatigue cracks around the fatigue-susceptible area (see Figure 1c). More details about distortion-induced fatigue cracks can be found in [31,32,33].

As the main mechanism for sensing the growth of distortion-induced fatigue cracks validated in the previous laboratory investigations [18,30] and adopted in the present study, the SEC sensors are applied to the web-gap region to capture the average capacitive strain, denoted by $\Delta C\left(t\right)$, under traffic loading. However, $\Delta C\left(t\right)$ alone is not adequate to monitor the crack growth because its value would change when the traffic load changes, even without any crack growth. Therefore, the out-of-plane force,  F(t), which is proportional to the traffic loading, is needed to normalize $\Delta C\left(t\right)$ and eliminate the influence of the traffic loading. In practice, F(t) can be obtained by measuring the strain of a member of the cross-frame using a strain gauge. It is important to clarify that the strain gauge is not meant to monitor the crack directly, but rather to indirectly capture the traffic loading for normalizing the large-area capacitive strain to enable reliable crack monitoring using the SECs.

According to the above crack-sensing mechanism, in the laboratory studies [18,30], the crack growth index, CGI, was proposed as the ratio between $\Delta C\left(t\right)$ and F(t). Because the traffic loading was simplified as a harmonic function, Fourier transform was used to obtain the magnitudes of both $\Delta C\left(t\right)$ and F(t) from noisy measurements. However, in practice, under real-world random traffic events, the field data of $F\left(t\right)$ and $\Delta C\left(t\right)$ are nonstationary signals consisting of unevenly distributed impulsive components, which are also contaminated by noise and low-frequency drift. As a result, the CGI based on the Fourier transform would no longer be able to extract reliable information for fatigue crack monitoring. To address this challenge, this study proposed a modified CGI based on the Wavelet transform to process real-world nonstationary traffic-induced bridge response signals. In addition, the WLASS is developed to replace the wired data acquisition system used in the laboratory studies, enabling long-term autonomous fatigue crack monitoring in the field. In the next section, the main methodology is presented, including both the hardware and algorithm aspect of the developed WLASS.

## 3. Methodology

### 3.1. The WLASS: Hardware

The WLASS is proposed by integrating: (1) SEC to measure large-area strain; (2) the Xnode wireless smart sensing platform for autonomous trigger-based sensing, wireless data collection, cloud storage, and remote data retrieval; and (3) capacitive sensor board and DC Wheatstone bridge to interface both the SEC and a point sensor, strain gauge, with the Xnode for measuring the signals of $\Delta C\left(t\right)$ and $F\left(t\right)$, respectively.

#### 3.1.1. SEC for Large-Area Strain Sensing

**Figure 2:**
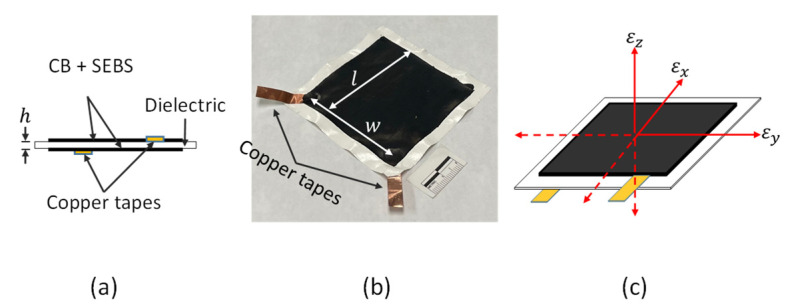
Illustration of the SEC sensor with the principal axes of strains: (**a**) structure of SEC, (**b**) picture of an SEC, and (**c**) SEC with the principal axes of strains.

The core element of the WLASS is the SEC which transduces large-area in-plane strain into a change in capacitance, $\Delta C\left(t\right)$ [20]. Compared with other conventional resistive-type strain sensors, the SEC is highly stretchable and hence can effectively monitor crack growth without being damaged under crack opening and closing. Other significant advantages include its ability to cover a large area, high conformability due to its high stretchability and flexibility, and low cost. The SEC consists of a dielectric layer sandwiched between two conductive layers as electrodes. The dielectric layer contains the block co-polymer styrene-ethylene/butylene-styrene (SEBS) doped with titania (TiO2) (Figure 2a). The conductive layers are also constructed from a SEBS matrix but doped with conductive carbon black (CB). The electrical model of the SEC is formulated as:

(1) $$C=\frac{e_{0}e_{r}A}{h}$$

where A is the sensing area of the SEC, equal to w×l, with w and l being the width and length of the sensing area, respectively; h is the thickness of the dielectric, which is around 0.22 mm in this study; $e_{0}$ is the vacuum permittivity; and $e_{r}$ is the dielectric permittivity. More detailed descriptions of the SEC can be found in [20]. In this paper, the SECs of size 76.2 mm × 76.2 mm are considered.

The SECs are installed on structural surfaces using a commercial two-part epoxy, J-B weld. As shown in Figure 2b, two copper tapes are attached to the bottom and top conductive layers to connect the SEC with data acquisition to measure large-area in-plane strain in terms of capacitance, $\Delta C\left(t\right)$. In particular, when installed over fatigue cracks, the opening and closing of the crack leads to changes in the SEC’s geometry, A and h, resulting in capacitance change, $\Delta C\left(t\right)$. Differentiating Equation (1) and assuming small changes in geometry, a mathematical expression for ΔC can be obtained in relation with strain [29]:

(2) $$\frac{\Delta C}{C}=\frac{\Delta w}{w}+\frac{\Delta l}{l}-\frac{\Delta h}{h}=\varepsilon_{x}+\varepsilon_{y}-\varepsilon_{z}$$

where $\varepsilon_{x},\;\varepsilon_{y},\;\mathrm{and}\;\varepsilon_{z}$ are the average strains in x, y, and z axes (see Figure 2c), respectively. Since the capacitance change in the SEC is only related to the deformation of the dielectric layer, which can be considered isotropic, the stress along the z-axis can be formulated under Hooke’s law using the plane stress assumption:

(3) $$\varepsilon_{z}=-\frac{v}{1-v}\left(\varepsilon_{x}+\varepsilon_{y}\right)$$

Subsequently, by combining Equations (2) and (3), one can obtain:

(4) $$\frac{\Delta C}{C}=\frac{1}{1-v}\left(\varepsilon_{x}+\varepsilon_{y}\right)$$

or,

(5) $$\Delta C=\frac{1}{1-v}\left(\varepsilon_{x}+\varepsilon_{y}\right)\;C$$

where $\frac{1}{1-v}$ can be treated as a gauge factor, and v is the Poisson ratio of the dielectric [29]. Equation (5) indicates that ΔC has a linear relationship with the total in-plane strain under the SEC sensor. Thus, ΔC is used here to represent the total in-plane strain in unit of pF, which is measured using the proposed WLASS explained below. Note that this research relies on measuring the total in-plane strain, which consists of strain in both *x* and y directions, to infer crack growth under a single SEC sensor, while crack propagation direction can be monitored using a network of SECs, as validated experimentally in [30].

#### 3.1.2. The Data Acquisition System for WLASS

**Figure 3:**
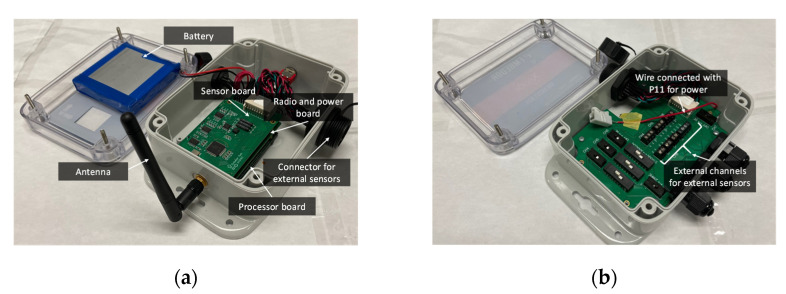
The Xnode wireless smart sensor platform: (**a**) the Xnode sensor node and (**b**) breakout box.

This study aims at enabling wireless sensing with the large-area strain sensor, SEC, for long-term fatigue crack monitoring in field applications. To this end, as mentioned previously, the Xnode wireless smart sensor platform [8,11,12] developed for SHM applications was selected for its flexible interface with external sensors, reliable wireless communication, high sampling rate, sensing resolution, and rugged design. As shown in Figure 3a, the Xnode sensor consists of three boards, including a sensor board, a radio and power board, and a processor board, as well as a lithium-ion battery which can be charged using a solar panel. The radio board, along with the antenna, offers a line-of-sight communication distance of 3 km [34]. To enable event-triggered sensing mode, a lower-power trigger accelerometer, ADXL362 by Analog Devices, was adopted in the Xnode [12]. Through a predefined acceleration threshold, an event-triggered sensing mode can be used to only measure significant vibration events, resulting in improved power efficiency for long-term fatigue crack monitoring. The Xnode sensor network contains sensor nodes responsible for sensing and one gateway node that communicates with PC and sensor nodes to receive the measured data. In this study, the cellular gateway node equipped with 4G-LTE modem for data transmission, cloud storage, and remote data retrieval developed in [11] is utilized. In addition, the Xnode sensor is equipped with a 24-bit AD converter with eight sensing channels. The onboard tri-axial accelerometer uses the first three channels to measure accelerations in x, y, and z directions, while the remaining five channels can be used to measure analog voltage signals from external sensors such as strain gauges, including the SEC used in this study. Thus, to ensure robust connections, a breakout box shown in Figure 3b is used to connect the SECs to the Xnode through the connectors.

**Figure 4:**
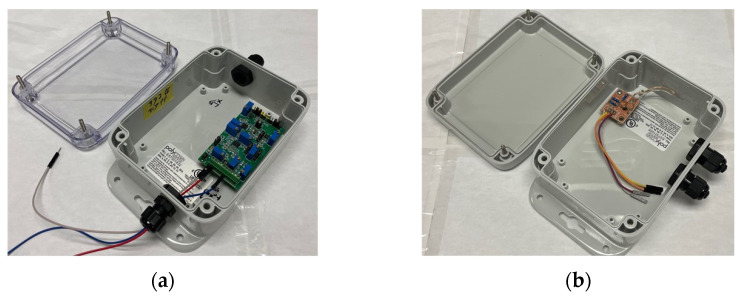
(**a**) The capacitive sensor board and (**b**) the Wheatstone bridge packaged in a weatherproof enclosure.

The five extra channels of the Xnode accept analog voltage signals ranging from 0 to 2.4 V. To achieve wireless sensing for the SEC, as shown in Figure 4a, a capacitive sensor board [21] that converts the capacitive large-area strain signal, $\Delta C\left(t\right)$, from the SEC to voltage signal was developed, which enables using the SEC with the Xnode wireless sensing platform and enhancing the quality of large-area wireless strain measurement [21]. The capacitive sensor board was designed to use the 3.3 V power supply from the Xnode and receive the $C\left(t\right)$ signal from SEC and convert it to a measurable change in voltage, which falls within the input voltage range (0–2.4 V) of the external channels. Moreover, a two-step shunt calibration process was included in the capacitive sensor board to carry out accurate and robust onboard calibration for capacitance measurement. More details about the capacitive sensor board can be found in [21]. In this research, the capacitive sensor board is packaged in a weather-proof enclosure, as shown in Figure 4a.

The proposed algorithm which will be discussed in the next section requires the information of the $\Delta C\left(t\right)$ normalized by $F\left(t\right)$ as discussed in Section 2. Thus, the out-of-plane force, $F\left(t\right)$, of the cross-frame is indirectly captured using a foil-type strain gauge. To enable wireless strain sensing with the external analog channels of the Xnode, a DC (direct current) Wheatstone bridge [35] shown in Figure 4b is used to indirectly measure the out-of-plane force, $F\left(t\right)$, of the web-gap region exerted by the cross-frame. The output signal obtained from the Wheatstone bridge is in voltage. Thus, the following equation is used to convert the voltage signal to strain [35]:

(6) $$\Delta V=\frac{GF\times \varepsilon }{4}V_{EXT}$$

where ΔV is the output voltage, GF is the gauge factor of the strain gauge, ε  is the strain to indirectly capture the out-of-plane force $F\left(t\right)$, and $V_{EXT}$ is the excitation voltage. Here, since the Wheatstone bridge is also powered by the Xnode, the $V_{EXT}$ is equal to 3.3 V. The Wheatstone bridge is also packaged in a weather-proof enclosure (see Figure 4b).

**Figure 5:**
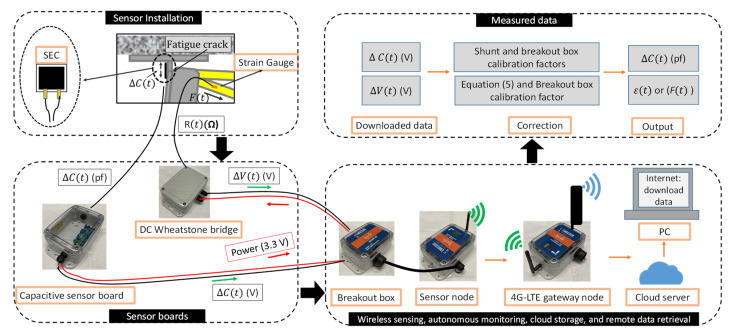
The proposed WLASS for wireless sensing, cloud storage, and remote data retrieval for fatigue crack monitoring.

The proposed WLASS is summarized in Figure 5. As illustrated in the figure, the $\Delta C\left(t\right)$ and $F\left(t\right)$ signals are measured by the SECs and strain gauge through the capacitive sensor board and DC Wheatstone bridge, respectively. The sensor node equipped with the breakout box supplies the 3.3 V power to the capacitive sensor board and the DC Wheatstone bridge, which return voltage signals for the $\Delta C\left(t\right)$ and $F\left(t\right)$, respectively, to the sensor node. The cellular gateway node communicates with the sensor node to receive measured signals and upload them to the cloud server. The cloud data can be accessed using a PC through the internet and a web browser. Finally, $\Delta C\left(t\right)$ and $F\left(t\right)$ are obtained by first applying the breakout box factor, followed by the shunt calibration coefficient and Equation (4), respectively.

### 3.2. The WLASS: Algorithm for Fatigue Crack Monitoring

The proposed algorithm for fatigue crack monitoring is discussed here. It contains three steps, automated traffic event detection, a generalized Morse continuous wavelet transform (GM-CWT) and peak identification, and a modified CGI, to compute the modified CGI in presence of non-stationary signal with numerous impulsive peak events due to traffic loading, as explained below.

The premise of the proposed algorithm for fatigue crack monitoring is that the initiation and propagation of fatigue cracks introduce an increase in the local strain response around the cracked region, causing it to be out of proportion to the applied loading. In other words, once normalized against the loading, the local strain response would increase if a new crack is developed or an existing crack propagates further. As a result, identifying the amplitudes of the large-area strain, $\Delta C\left(t\right)$, and the strain-based, indirectly measured out-of-plane force, $F\left(t\right)$, under traffic loading is critical to successful crack growth monitoring (see Figure 1). In particular, the amplitude of $\Delta C\left(t\right)$ is normalized by *F*$\left(t\right)$ to remove the influence of the changing load amplitude, leading to the CGI.

**Figure 6:**
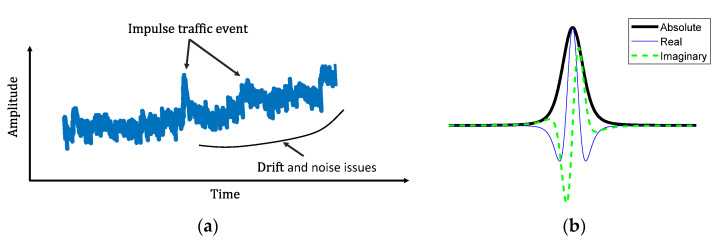
(**a**) A typical signal under traffic loading using the WLASS, and (**b**) The shape of the generalized Morse Wavelet with γ = 1.5 and $P^{2}=3$.

As shown in Figure 6a, both *F*(*t*) and $\Delta C\left(t\right)$ data are nonstationary signals and contain impulsive components due to traffic events, as well as noise and low-frequency drift. As mentioned previously, extracting the amplitude information from those signals based on Fourier transform and calculating the CGI is challenging. In this study, the continuous wavelet transform (CWT) designed for processing non-stationary signals [36] is adopted to reliably extract the amplitudes corresponding to the impulsive loading events, based on which the modified CGI is proposed. The CWT decomposes a signal into the time-frequency domain, which enables an accurate characterization of the signal’s changing energy level over time at various frequencies. The mathematical formula of the CWT for signal $x\left(t\right)$ is expressed as [36,37]:

(7) $$W\left(t,s\right)=\int_{-\infty }^{\infty }\frac{1}{s}\varphi^{*}\left(\frac{\tau -t}{s}\right)\;x\left(\tau \right)\;d\tau$$

where $W\left(t,s\right)$ is a matrix with complex values that carries information about the amplitudes of wavelet coefficients, φ is the wavelet function, in which the asterisk denotes the complex conjugate, and t and s are time and scale parameters, respectively. Note that $x\left(t\right)$ can be $\Delta C\left(t\right)$ or $F\left(t\right)$ in this paper. Time-frequency analysis with the CWT for non-stationary signals depends on the wavelet function φ. To this end, the generalized Morse wavelets for CWT (GM-CWT) proposed in [36,37,38] are chosen for their ability to imply various analytic wavelets by adjusting their parameters, which are formulated as [36,37]:

(8) $$\varphi_{P,\gamma }\left(\omega \right)=U\left(\omega \right)\;a_{P,\gamma }\;\omega^{\frac{P^{2}}{\gamma }}e^{-\omega^{\gamma }}$$

where $U\left(\omega \right)\;$ is the Heaviside step function, γ and $P^{2}$ are the generalized Morse wavelet parameters to control the symmetry and the oscillations of the wavelet, and $a_{P,\gamma }$ is a normalizing constant. By adjusting both (γ, $P^{2}$) parameters, various shapes of wavelets can be achieved. Specifically, to extract the amplitudes for the impulse events due to traffic loading, γ = 1.5 and $P^{2}=3$ are chosen in this paper. The shape of the wavelet based on the chosen parameters is shown in Figure 6b. The Wavelet toolbox in the MATLAB R2021a program was used in this paper, in which L1 normalization was utilized for a more accurate representation of the signal [39]. Compared to other shapes of wavelets, such as the Morlet wavelet, Bessel wavelet, Cauchy wavelets, etc., the selected shape can effectively separate and extract the amplitude information of impulse traffic events. The proposed algorithm is summarized in the following steps:

Step 1. Automated traffic event detection: The impulse traffic events are automatically detected from $F\left(t\right)$ to obtain the times when the peak strain events occur. Define $I=[I_{1},I_{2},\ldots ,I_{n}$], $F=[F_{1},F_{2},\ldots ,F_{n}$], and $t=[t_{1},t_{2},\ldots ,t_{n}$], in which F contains the values of the detected peaks, t has the times associated with the peaks, I includes the indices of the detected peaks, and n is the total number of detected peaks. First, the local maxima of time history are identified based on the change of derivatives. Then, the local maxima that exceed a pre-defined threshold value, h, are retained as the detected peaks. In this study, the $F\left(t\right)$ threshold of h=30 με is selected as the threshold to ensure large enough events are kept. In addition, a minimum peak distance of $t_{d}$ is also implemented to avoid closely spaced peaks, in which a value between 1 s and 2 s is recommended for traffic loads. Here, $t_{d}=1.3\;s$ is assumed in this study.

Step 2. GM-CWT and peak identification: using the proposed GM-CWT, ${\left|W\left(t,s\right)\right|}_{C}$ and ${\left|W\left(t,s\right)\right|}_{F}$ are computed for $\Delta C\left(t\right)$ and $F\left(t\right)$ signals, respectively. To identify robust peak values embedded in ${\left|W\left(t,s\right)\right|}_{C}$ and ${\left|W\left(t,s\right)\right|}_{F}$ associated with traffic events for computing the modified CGIs, windows of interest (WOI) based on the detected traffic events in Step 1 are first defined as ${\mathrm{WOI}}_{i}$ = [$t_{i}-t_{d}$, $t_{i}+t_{d}$], where i=1, 2,…,n. Subsequently, ${\left|W\left(t,s\right)\right|}_{C_{i}}^{\max}$ and ${\left|W\left(t,s\right)\right|}_{F_{i}}^{\max}$, which are the maximum values of ${\left|W\left(t,s\right)\right|}_{C}$ and ${\left|W\left(t,s\right)\right|}_{F}$, respectively, associated with the detected traffic events are obtained within each ${\mathrm{WOI}}_{i}$. Note that ${\left|W\left(t,s\right)\right|}_{C_{i}}^{\max}$ is obtained using two cases. In Case-1, ${\left|W\left(t,s\right)\right|}_{C_{i}}^{\max}$ is computed using the index of ${\left|W\left(t,s\right)\right|}_{F_{i}}^{\max}$, the location of ${\left|W\left(t,s\right)\right|}_{F_{i}}^{\max}$, by assuming the peak events of $\Delta C\left(t\right)$ and $F\left(t\right)$ occur at the same time, while ${\left|W\left(t,s\right)\right|}_{C_{i}}^{\max}$ for Case-2 is calculated based on the maximum values of ${\left|W\left(t,s\right)\right|}_{C}$ within the ${\mathrm{WOI}}_{i}$.

Step 3. The modified CGI: The modified CGI which is an indicator for monitoring fatigue cracks is computed for each peak event as:

(9) $${\mathrm{CGI}}_{i}=\frac{{\left|W\left(t,s\right)\right|}_{C_{i}}^{\max}}{{\left|W\left(t,s\right)\right|}_{F_{i}}^{\max}}$$

**Figure 7:**
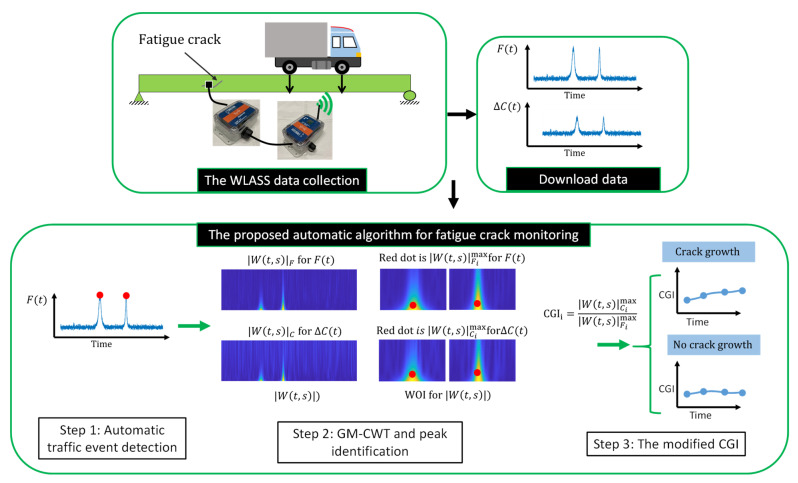
Workflow of the proposed automated algorithm for fatigue crack monitoring.

The CGI records the peak average strain under the SEC sensor normalized by the indirect out-of-plane force $F\left(t\right)$ under traffic loading. In this paper, the mean of ${\mathrm{CGI}}_{i}$, $\bar{\mathrm{CGI}}$, and the associated standard deviation based on impulsive traffic events recorded in one to two days with a minimum of two events are computed to show the results of the crack monitoring. Potential crack initiation and propagation are represented by changes in $\bar{\mathrm{CGI}}$. As illustrated in Figure 7, $\Delta C\left(t\right)$ and $F\left(t\right)\;$ are obtained from the WLASS under traffic loading. Subsequently, the measured data are processed with the proposed algorithm for fatigue crack monitoring. Specifically, using $F\left(t\right)$, the impulsive traffic events are automatically detected through Step 1. Next, using the proposed GM-CWT in Step 2, ${\left|W\left(t,s\right)\right|}_{C}$ and ${\left|W\left(t,s\right)\right|}_{F}$ are computed for $\Delta C\left(t\right)$ and $F\left(t\right)$, respectively. Consequently, the WOIs for ${\left|W\left(t,s\right)\right|}_{C}$ and ${\left|W\left(t,s\right)\right|}_{F}$ are identified based on the peak detections in Step 1, and the peak amplitudes, ${\left|W\left(t,s\right)\right|}_{C_{i}}^{\max}$ and ${\left|W\left(t,s\right)\right|}_{F_{i}}^{\max}$ are estimated within the WOIs. Finally, in Step 3, the modified CGIs are computed based on ${\left|W\left(t,s\right)\right|}_{C_{i}}^{\max}$ and ${\left|W\left(t,s\right)\right|}_{F_{i}}^{\max}$ using Equation (8) from Step 3 to monitor crack growth. As illustrated in Figure 7, if the crack grows, the CGI would gradually increase. Otherwise, the CGI remains constant if there is no crack growth.

## 4. Numerical Validation for the GM-CWT Algorithm

**Figure 8:**
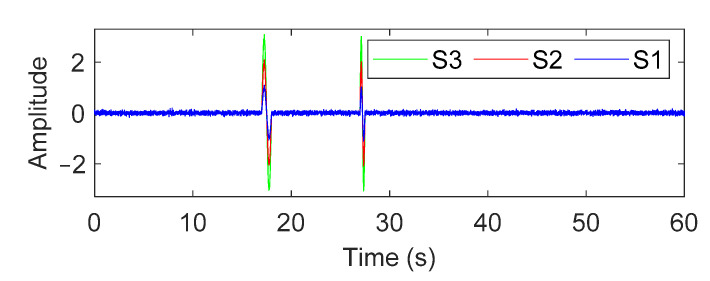
The simulated traffic-induced impulsive signal for all three scenarios with 30% measurement noise.

In this section, the performance of the GM-CWT algorithm is numerically validated for extracting the amplitude information of a nonstationary signal that contains discrete impulsive events. The numerical example considers a simulated traffic-induced impulsive signal that consists of two cycles of a sine wave with three scenarios listed in Table 1. For all three scenarios, the frequencies of the first and second cycles are equal to 1 Hz and 2 Hz, respectively, while each scenario has different amplitudes for the sine waves. To consider measurement noise in practice, 30% zero-mean Gaussian white noise is added to the signals, as shown in Figure 8.

**Figure 9:**
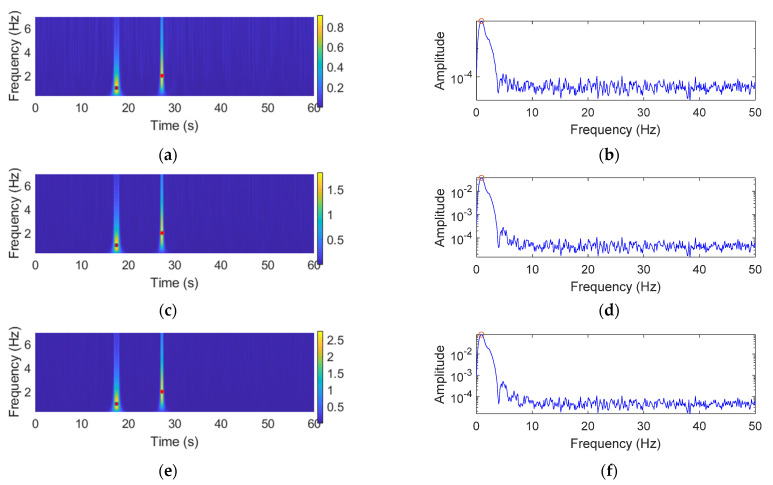
Signal processing results using GM-CWT and Auto PSD. The $\left|W\left(t,s\right)\right|:\;$(**a**) S1, (**c**) S2, and (**e**) S3. The auto PSDs: (**b**) S1, (**d**) S2, and (**f**) S3.

To compare the results with the traditional Fourier-analysis-based method [18], in addition to calculating the magnitudes of the wavelet coefficients, $\left|W\left(t,s\right)\right|$, using the GM-CWT algorithm, the auto power spectral densities (PSDs) which are based on the Fourier transform are also computed for the signals. Figure 9 shows the $\left|W\left(t,s\right)\right|$ and auto PSDs for all three scenarios. Note that two red dots in Figure 9a,c,e depict the frequencies and times of the identified peaks of the two impulsive events. As summarized in Table 2, the GM-CWT algorithm achieved high accuracies in identifying frequency, time, and amplitude for both impulsive events in all three scenarios. Note that the extracted amplitudes obtained from the GM-CWT are close to the true ones for this particular signal since the L1 normalization was used in GM-CWT, as explained previously in Section 3.2. The result validates the capability of the GM-CWT algorithm in extracting and separating the amplitude information of the simulated traffic-induced response signal in the time-frequency domain. On the other hand, the PSD plots are shown in Figure 9b,d,f only return a single peak for each signal due to the fact that Fourier transform assumes the signal is stationary and produces a temporarily-averaged energy distribution in the frequency domain. The extracted amplitudes and frequencies obtained from the PSDs are indicated in Table 2, which are shown as small circles in Figure 9b,d,f. Overall, compared with PSD, the GM-CWT extracted the impulsive events in all three scenarios with high accuracies in terms of frequencies, times, and amplitudes. The result validated numerically that the proposed GM-CWT in Step 2 of the proposed algorithm is able to extract accurate amplitude information of the impulsive traffic events from the nonstationary signals for computing the modified CGI in Step 3.

## 5. Field Validation for Fatigue Crack Monitoring

**Figure 10:**
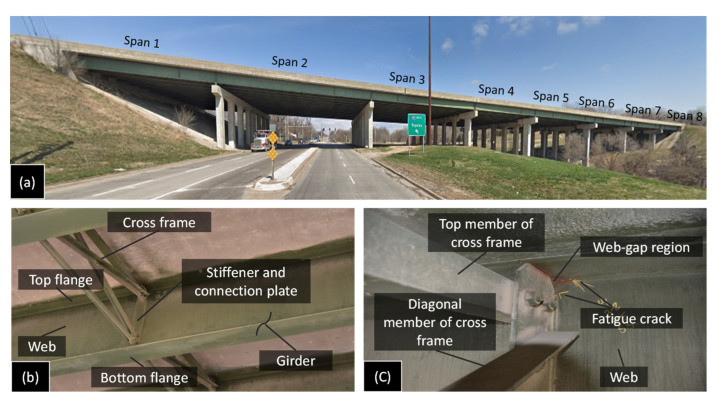
I-70 highway bridge: (**a**) span layout of the bridge, (**b**) cross-frame between the adjacent girders, and (**c**) detail of the web-gap region with distortion-induced fatigue cracks.

The performance of the proposed WLASS and automated algorithm for fatigue crack monitoring are examined using a field bridge. As shown in Figure 10a, the field validation was carried out on a steel highway bridge, designated the 70-105-41732-128 (eastbound) bridge on the I-70 highway near Kansas City, Kansas, located at the intersection with N. 57^th^ Street. According to the inspection reports by the Kansas Department of Transportation (KDOT), multiple locations on the bridge are subjected to fatigue damage and have existing fatigue cracks, which are mainly distortion-induced fatigue cracks located at the cross-frame-to-girder connections and the web-gap regions. Figure 10c shows two sample fatigue cracks in the bridge, with one located at the web-gap region along the weld between the cross frame and girder-to-web connection plate and another one along the weld toe between the girder flange and web. As discussed previously in Section 2, these cracks are caused by the differential movement between the two adjacent girders, which leads to the out-of-plane force on the girder web by the cross-frame, resulting in the initiation and propagation of the fatigue cracks.

### 5.1. Selection of a Fatigue Crack Location and the WLASS Installation

**Figure 11:**
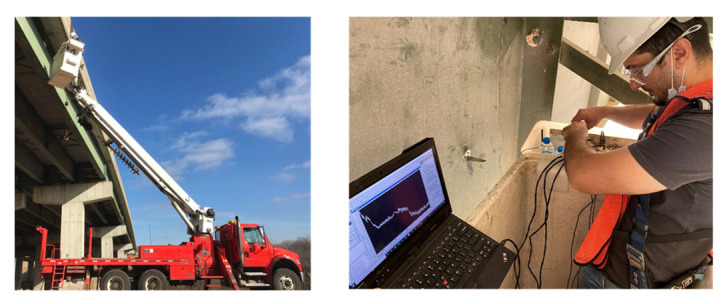
Man-lift truck to access the regions with cracks in the bridge girders.

**Figure 12:**
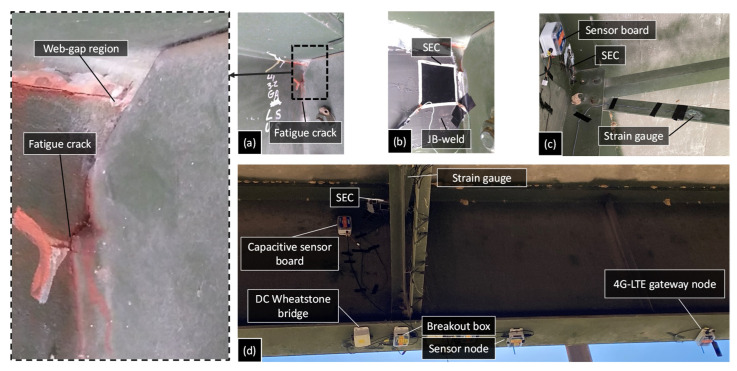
Installation of the proposed WLASS: (**a**) figure crack, (**b**) SEC, (**c**) strain gauge, and (**d**) installation of the WLASS.

The selection of a fatigue crack location and WLASS installation are discussed here. A man-lift truck was used to facilitate access to the steel girders and the WLASS installation (see Figure 11). The interior side of the exterior girder within Span 3 shown in Figure 10a was chosen for field validation. In the steel girder, a transverse connection plate connects the cross-frame to the girder web. A distortion-induced fatigue crack due to the differential movement between the two adjacent girders has been growing around the web-gap region along the weld between the transverse connection plate and the girder web, which has also propagated into the web, as shown in Figure 12a. Therefore, this distortion-induced fatigue crack region and the diagonal member of the cross-frame were instrumented with the WLASS as illustrated in Figure 12. Particularly, a large-area strain sensor, SEC, was installed on the girder web to cover the fatigue crack and capture the average strain,$\;\Delta C\left(t\right)$, in the cracked region. Meanwhile, a strain gauge was attached to one diagonal member of the cross frame to indirectly measure the out-of-plane force, $F\left(t\right)$. The structural surfaces were sanded to remove paint and cleaned up for installing the SEC and the strain gauge. A two-part epoxy J-B weld was used to install the SEC, while adhesive and coating were applied to install the strain gauge on the cross frame (see Figure 12b,c). Subsequently, other components of the WLASS, including the capacitive sensor board, DC Wheatstone Bridge, breakout box, wireless sensor node, and the 4G cellular gateway were installed based on the schematic described in Figure 5. Figure 12d provides an overview of the WLASS installation on the steel bridge. The capacitive sensor board was installed close to the SEC to minimize the cable length and hence noise for capacitance measurement, while the DC Wheatstone Bridge, breakout box, sensor node, and 4G cellular gateway were attached to the bottom flange as shown in the figure. After completing the installation, AC Bridge balancing and Shunt calibration was performed for the capacitive sensor board of the SEC (see Figure 11). Finally, the WLASS was ready to collect data of $\Delta C\left(t\right)$ and $F\left(t\right)$ (see Figure 5 for more details).

### 5.2. Data Collection and Processing

**Figure 13:**
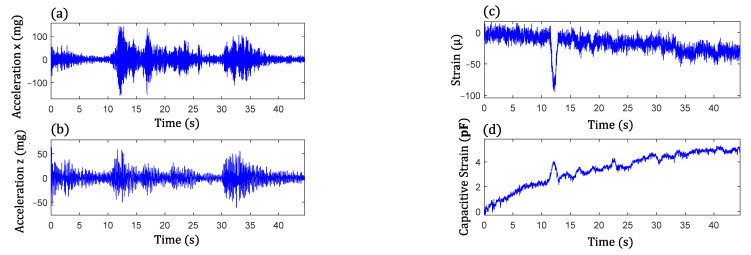
A sample data set from the WLASS including: (**a**) lateral acceleration and (**b**) vertical acceleration; (**c**) cross-frame strain, $F\left(t\right)$; and (**d**) large-area strain, $\Delta C\left(t\right)$.

To enable energy-efficient long-term monitoring, the event-triggered sensing mode discussed in Section 3.1 was used to capture significant traffic loading events and collect meaningful data using the WLASS through a predefined triggering acceleration threshold. In particular, event-triggered sensing was based on the acceleration response at the bottom flange where the sensor node was installed. After monitoring the vibration levels of the bottom flange due to traffic loading for a short period of time, acceleration thresholds between 150 mg and 250 mg were used, such that only significant loading events were measured for long-term fatigue crack monitoring. Figure 13 shows an example of raw acceleration measurements in lateral (x) and vertical (z) directions, large-area strain,$\;\Delta C\left(t\right)$, and cross-frame strain, $F\left(t\right)$. The acceleration measurements indicate several traffic loading events through high acceleration levels. The acceleration in the longitudinal direction (y) is not shown here since it has a much lower level of vibration compared to other directions. From Figure 13c,d, a large impulsive event due to the traffic loading can be seen clearly between 11 s and 13 s, which also matches with the first peak acceleration event in both the lateral and vertical directions. Note that the acceleration response of the girder flange in the lateral direction dominated this event with a peak acceleration of around 150 mg, which is also the case for the cross-frame strain $F\left(t\right)$, and $\Delta C\left(t\right)$ as the first peak dominated the measurement.

### 5.3. The GW-CWT Results

**Figure 14:**
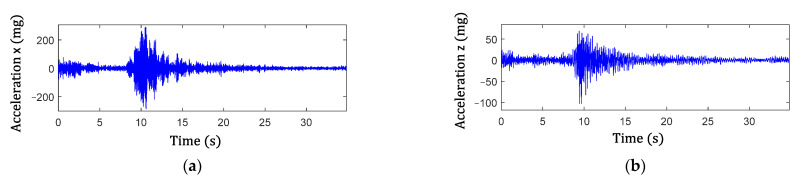
Acceleration measurements containing single impulsive traffic event: (**a**) lateral direction and (**b**) vertical direction.

**Figure 15:**
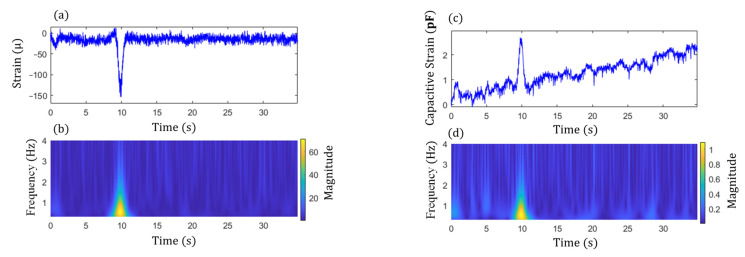
Single impulse traffic event: (**a**) raw data and (**b**) magnitude, ${\left|W\left(t,s\right)\right|}_{F}$, of cross-frame strain, $F\left(t\right)$, and (**c**) raw data and (**d**) magnitude, ${\left|W\left(t,s\right)\right|}_{C}$, of large-area strain, $\Delta C\left(t\right)$.

**Figure 16:**
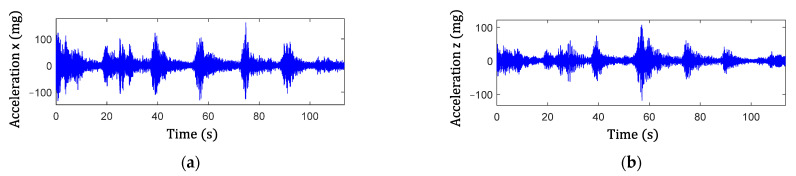
Acceleration measurements containing multiple impulse traffic events: (**a**) lateral direction and (**b**) vertical direction.

**Figure 17:**
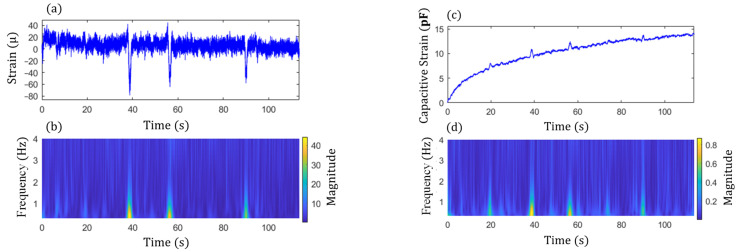
Multiple impulse traffic events: (**a**) raw data and (**b**) magnitude, ${\left|W\left(t,s\right)\right|}_{F}$, of cross-frame strain, $F\left(t\right)$, and (**c**) raw data and (**d**) magnitude, ${\left|W\left(t,s\right)\right|}_{C}$, of large-area strain, $\Delta C\left(t\right)$.

Step 2 in the proposed algorithm is to extract the signal components of the peak events and is illustrated here using two examples. The examples consider both single and multiple traffic events. Figure 14 shows the acceleration responses in the lateral and vertical directions for single traffic event. The high amplitude accelerations in the data are due to the traffic loading. Figure 15a,c show the associated cross-frame strain measurement, $F\left(t\right)$, and large-area strain, $\Delta C\left(t\right)$, signals, respectively. For multiple traffic events, the corresponding measurements are shown in Figure 16 and Figure 17a,c. The impulsive events due to the traffic loadings are observed from the signals. Moreover, the impulse events again match with the high amplitude oscillation events of the accelerations for both the single and multiple traffic events. Note that, as shown in the figures, both $F\left(t\right)$ and $\Delta C\left(t\right)$ are nonstationary and have noise, and $\Delta C\left(t\right)$ has significant low-frequency drifts.

Subsequently, the magnitudes of GM-CWT described in Step 2 of the proposed algorithm, ${\left|W\left(t,s\right)\right|}_{c}$ and ${\left|W\left(t,s\right)\right|}_{F}$, were calculated for $\Delta C\left(t\right)$ and $F\left(t\right)$ for both examples, respectively. The sampling rate was 100 Hz, and the lower and upper cutoff frequencies were chosen as 0.3 Hz and 4 Hz, respectively, for the GM-CWT to remove high-frequency noise and low-frequency drift. As mentioned previously, GM-CWT parameters of γ = 1.5 and $P^{2}=3$ were chosen to control the symmetry and the oscillations of the wavelet in this paper. The results of ${\left|W\left(t,s\right)\right|}_{F}$ and ${\left|W\left(t,s\right)\right|}_{C}$ under the single and multiple traffic, events are shown in Figure 15b,d and Figure 17b,d, respectively. The hot spots in the figures indicate the extracted amplitude information in both the time and frequency domains, which are associated with impulsive traffic events. The second part of Step 2 is to extract the amplitude information from ${\left|W\left(t,s\right)\right|}_{C}$ and ${\left|W\left(t,s\right)\right|}_{F}$ using the WOIs, which will be described in the next section.

### 5.4. Fatigue Crack Monitoring Results

#### 5.4.1. Automated Traffic Event Detection (Step 1) and Peak Identification (Step 2)

**Figure 18:**
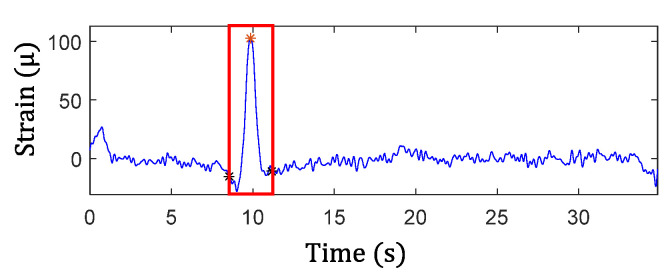
Identified traffic events and WOIs from strain, $F\left(t\right)$, measurement under a single traffic event.

**Figure 19:**
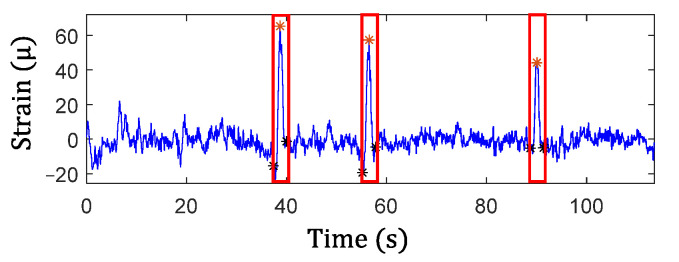
Identified traffic events and WOIs from strain, $F\left(t\right)$, measurement under multiple traffic events.

The results from Step 1 for automated traffic event detection and Step 2 for peak identification in the proposed algorithm are discussed here. Automated traffic event detection in Step 1 was performed based on the measured cross-frame strain, $F\left(t\right)$, to identify impulsive traffic events for robustly extracting the amplitudes for the GM-CWT results. The cross-frame strain, $F\left(t\right)$, was used for this purpose because it indirectly captures the traffic loading and has relatively low noise, hence more clear peaks. Subsequently, the peak detection results were used in Step 2 to find the WOIs for effectively extracting peaks in ${\left|W\left(t,s\right)\right|}_{C}$ and ${\left|W\left(t,s\right)\right|}_{F}$ and computing the modified CGI. As mentioned previously, the cross-frame strain has noise and low-frequency drifts, as shown in Figure 15a and Figure 17a. Thus, detrending, high-pass, and low-pass filtering were first utilized to remove the low-frequency drift and high-frequency noise in the $F\left(t\right)$ signal prior to peak detection. Utilizing the strain threshold of h=30 με and minimum peak distance of $t_{d}=1.3\;s$ defined in Section 3.2, the peaks were automatically detected using Step 1 of the proposed algorithm. The filtered signals with the detected peaks (red asterisks) are shown in Figure 18 and Figure 19 for both data sets.

**Figure 20:**
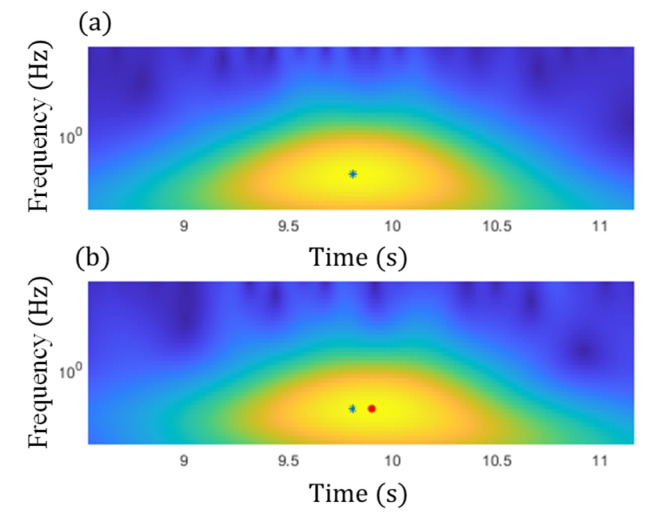
Peak identification within the identified WOIs under the single traffic event for (**a**) strain, $F\left(t\right)$, measurement, and (**b**) $\Delta C\left(t\right)$ from SEC.

**Figure 21:**
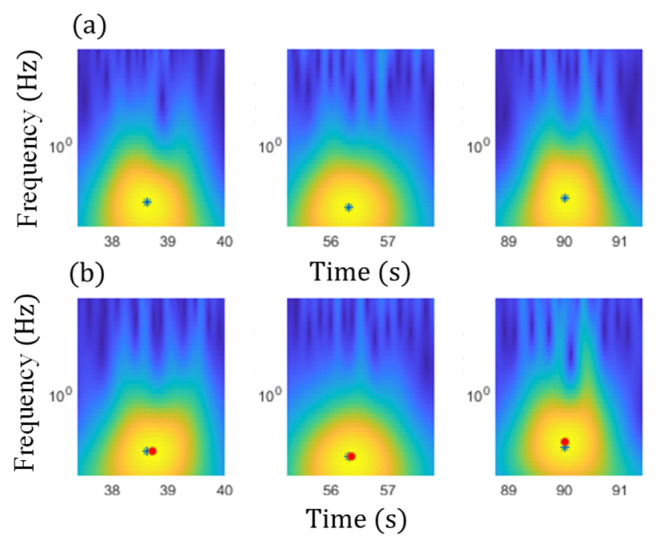
Peak identification within the identified WOIs under the multiple traffic events for (**a**) strain, $F\left(t\right)$, measurement, and (**b**) $\Delta C\left(t\right)$ from SEC.

Subsequently, the WOIs in Step 2 of the proposed algorithm was achieved as [$t_{i}-t_{d}$, $t_{i}+t_{d}$] using the timestamps of the detected peaks, $t_{i}$, which are shown in the figures by the black asterisks as well as the rectangular red boxes. The identified WOIs were then applied to the ${\left|W\left(t,s\right)\right|}_{C}$ and ${\left|W\left(t,s\right)\right|}_{F}$ calculated in the previous section to separate the impulsive traffic events and effectively extract ${\left|W\left(t,s\right)\right|}_{C_{i}}^{\max}$ and ${\left|W\left(t,s\right)\right|}_{F_{i}}^{\max}$ for each event, avoiding the impact of noise and low-frequency drift. The identified WOIs of the ${\left|W\left(t,s\right)\right|}_{C}$ and ${\left|W\left(t,s\right)\right|}_{F}$ are shown in Figure 20 and Figure 21 for both the single and multiple traffic examples, respectively. Note that the large yellow highlighted regions in the WOIs show the successfully extracted traffic events for $\Delta C\left(t\right)\;$ and $F\left(t\right)$. Based on the WOIs, the ${\left|W\left(t,s\right)\right|}_{C_{i}}^{\max}$ and ${\left|W\left(t,s\right)\right|}_{F_{i}}^{\max}$ were then extracted within their respective ${\mathrm{WOI}}_{i}$, and the results are illustrated in the figures. In particular, the blue stars in Figure 20a and Figure 21a show the ${\left|W\left(t,s\right)\right|}_{F_{i}}^{\max}$, which are the maximum peaks in the cross-frame strain, while ${\left|W\left(t,s\right)\right|}_{C_{i}}^{\max}$ of $\Delta C\left(t\right)$ for Case-1 and Case-2 are shown in blue stars and red dots in Figure 20b and Figure 21b, respectively. As a result, the modified CGI can be computed for both cases, as discussed in the next section.

#### 5.4.2. The Modified CGI for Fatigue Crack Monitoring (Step 3)

**Figure 22:**
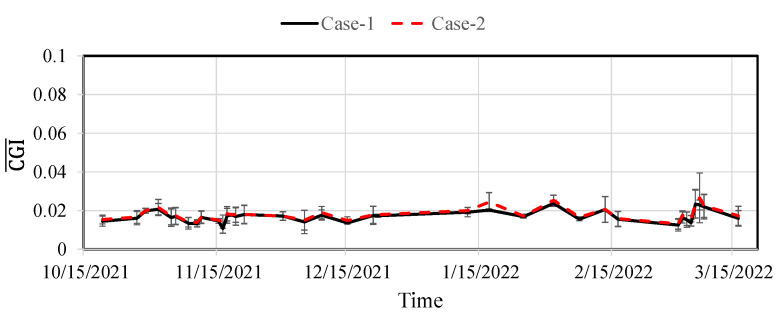
Monitored $\bar{\mathrm{CGI}}$ and standard deviation.

**Figure 23:**
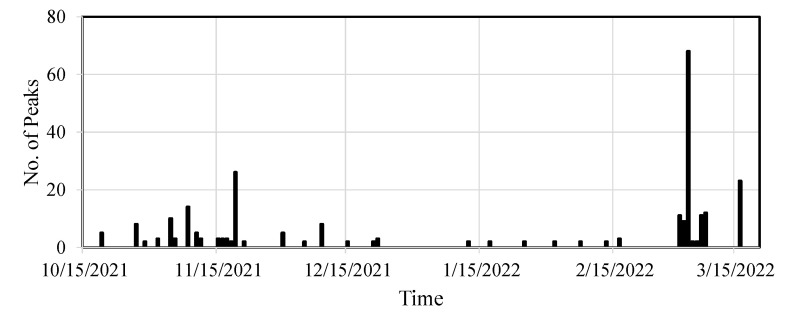
Number of peaks for computing the $\bar{\mathrm{CGI}}$.

The modified CGIs for the selected fatigue crack are presented here. Utilizing several acceleration thresholds ranging from 150 mg to 250 mg for event-triggered sensing, data from the bridge were collected using the WLASS from mid-October 2021 to mid-March 2022. A total of 129 datasets were used under the aforementioned strain threshold of h=30 με and minimum peak distance of $t_{d}=1.3\;s$ defined for peak detection in Step 1 to obtain the modified CGIs, which contain 267 impulse peak events. The modified CGIs for both cases were computed for each impulse peak event using Equation (8) based on the WOI. The identified CGIs over the monitoring period for both cases are shown in Figure 22. Note that each data point in the figure shows the mean modified CGI, $\bar{\mathrm{CGI}}$, and the associated standard deviation from impulsive traffic events collected from one to two days with a minimum of two events. For both cases, despite some fluctuation possibly attributed to sensor and data acquisition noise and limited data points, the $\bar{\mathrm{CGI}}$ stayed almost constant, indicating the crack size remained constant during the monitoring period. Moreover, the number of peaks for each $\bar{\mathrm{CGI}}$ data point in Figure 22 is shown in Figure 23. As shown in the figure, 19 November 2021, 4 March 2022, and 16 March 2022 recorded the highest number of peaks, including 26, 68, and 23 peaks, respectively. For Case-1, the corresponding $\bar{\mathrm{CGI}}$ are 0.017, 0.015, and 0.016, respectively, and the standard deviations are 0.0045, 0.004, and 0.004, respectively. For Case-2, the $\bar{\mathrm{CGI}}$ are 0.018, 0.015, and 0.017, respectively, and the standard deviations are 0.004, 0.0035, and 0.005, respectively. Note that those means and standard deviations remained almost constant, indicating no crack growth during the monitoring period.

### 5.5. Laboratory Investigation for the Results of the Modified CGI (Step 3) of the Bridge

**Figure 24:**
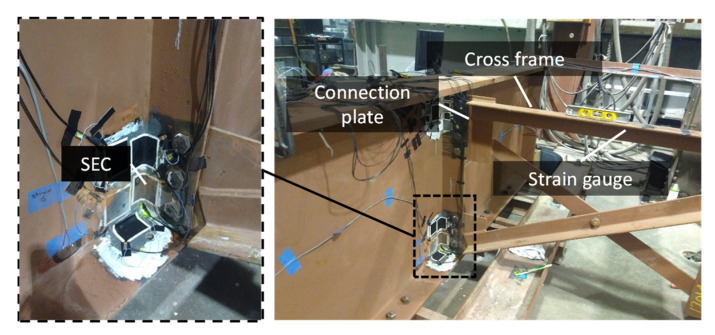
Laboratory non-skewed bridge girder test setup and the installed SECs and strain gauge [30].

Since the field result indicates no crack growth due to the constant $\bar{\mathrm{CGI}}$. To further understand the nature of the result shown in Figure 22, to serve as a control group, further laboratory investigation was carried out using a large-scale laboratory non-skewed bridge girder subassembly in [30], which is shown in Figure 24. A cross-frame was perpendicularly connected to the girder using a connection plate to mimic the case of the bridge in this paper. The fatigue load cycles were applied to the girder through an actuator installed vertically on one side of the cross-frame. The SECs and strain gauges were deployed to identify the traditional CGI for fatigue crack monitoring using a wired system. The traditional CGI requires the information on the large-area strain,$\;\Delta C\left(t\right)$, and the out-of-plane force, $F\left(t\right)$. Thus, as shown in Figure 24, an SEC was installed between the connection plate and the web to capture $\Delta C\left(t\right)$, while a strain gauge was attached to the top member of the cross-frame to measure the $F\left(t\right)$. The location was inspected to ensure there were no crack before installing the SEC. More details about the bridge girder and laboratory experiment can be found in [30].

**Figure 25:**
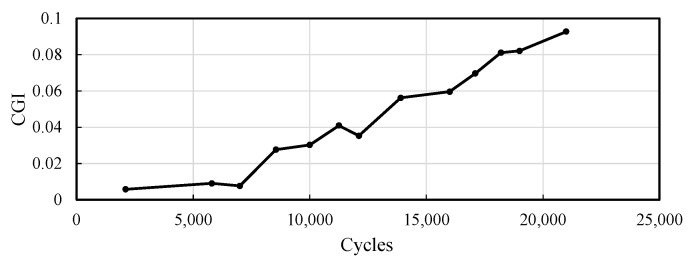
The results of monitored CGI presented [30] for the laboratory bridge girder setup.

A total of 18,900 fatigue load cycles with loads ranging between 2.2 kN and 25.5 kN were applied to the setup. At the end of the test, the SEC was removed, and a newly initiated fatigue crack was discovered through inspection after the test. In [30], the traditional CGI was calculated using 13 short time measurements of $\Delta C\left(t\right)$ and $F\left(t\right)$. After converting the units to match with the units used in this paper, the results of the CGI presented in [30] is shown in Figure 25. A gradual increase in the CGI can be seen with an increasing number of cycles showing successful fatigue crack monitoring. In particular, as shown in the figure, a first-initiated fatigue crack appeared at 8550 cycles, which resulted in a 478% change in CGI. However, the $\bar{\mathrm{CGI}}$ of the bridge shown in Figure 22 shows a much smaller level of fluctuation during the monitoring period. The level of change corresponding to actual crack growth observed in the laboratory test further verifies that no crack growth in the bridge according to the $\bar{\mathrm{CGI}}$ **was** obtained during the monitoring period.

## 6. Conclusions

This paper developed a wireless large-area strain sensor, WLASS, and its associated algorithm for fatigue crack monitoring. The WLASS integrates the SEC with the Xnode sensing platform to obtain large-area strain data wirelessly and operates under event-triggered sensing mode. In particular, the WLASS consists of (1) the SEC; (2) the Xnode wireless smart sensing platform for autonomous trigger-based sensing, wireless data collection, cloud storage, and remote data retrieval; and (3) a capacitive sensor board to interface the SEC with the Xnode for measuring large-area strain. Meanwhile, the developed automated algorithm copes with a large amount of non-stationary field data with numerous impulsive peak events due to traffic loads. The developed algorithm contains an automated traffic event detection, a GM-CWT and peak identification, and a modified CGI for fatigue crack monitoring. A numerical validation was conducted to examine the effectiveness of the GM-CWT of the developed algorithm to extract the peak traffic events. The results indicate that the GM-CWT is more suitable for analyzing nonstationary field traffic-induced signals compared with the traditional Fourier-analysis-based method. Subsequently, the performance of the developed WLASS and the algorithm was verified through a field deployment on a steel highway bridge in Kansas City, KS, USA. Data were collected from mid-October 2021 to mid-March 2022. Peak events due to traffic loadings were successfully identified, and the amplitude information was accurately extracted. Finally, the modified CGIs were obtained using the extracted amplitudes and were presented in terms of mean and standard deviation. As the highest number of daily peaks, 26, 68, and 23 impulsive events were obtained on 19 November 2021, 4 March 2022, and 16 March 2022, respectively. The corresponding $\bar{\mathrm{CGI}}$ were 0.017, 0.015, and 0.016 for Case-1 and 0.018, 0.015, and 0.017 for Case-2, respectively. The mean CGI remained relatively constant during the monitoring period, with a level of fluctuation much lower than that corresponds to actual crack growth observed in the control group based on a large-scale laboratory test, indicating that no crack propagation was detected during the relatively short monitoring period. Future work will be focused on long-term data analysis for fatigue crack monitoring using the WLASS.

## Figures and Tables

**Table 1:** Details of the simulated traffic-induced impulsive signals.

Scenario	Amplitude		Frequency (Hz)		Time (s)	
	First Cycle	Second Cycle	First Cycle	Second Cycle	First Cycle	Second Cycle
S1	1	1	1	2	17.5	27.25
S2	2	2	1	2	17.5	27.25
S3	3	3	1	2	17.5	27.25

**Table 2:** The results of the $\left|W\left(t,s\right)\right|$ and the Auto PSDs.

Scenario	|W(t,s)|						Auto PSD	
	Amplitude		Frequency (Hz)		Time (s)		Amplitude	Frequency (Hz)
	First Peak	Second Peak	First Peak	Second Peak	First Peak	Second Peak	Single Peak	Single Peak
S1	0.92	0.92	1.01	2.01	17.50	27.25	0.0093	0.88
S2	1.84	1.84	1.01	2.01	17.50	27.25	0.0372	0.88
S3	2.77	2.77	1.01	2.01	17.50	27.25	0.0839	0.88

## Data Availability

Data available on request.
